# Supplementary material for: Protective effects of combining monoclonal antibodies and vaccines against the Plasmodium falciparum circumsporozoite protein
Source: PLoS Pathog. 2021 Dec 6;17(12):e1010133. doi: 10.1371/journal.ppat.1010133 (PMC8675929; doi:10.1371/journal.ppat.1010133)
Supplement: S1 Table — mAb designation, original isotype (IgG vs. IgM; all mAbs used in this study were expressed as IgG1), species (human vs. mouse), target antigen, BLI apparent avidity for select PfCSP 15mer repeat peptides (peptide 21 NPDPNANPNVDPNAN, peptide 22 NANPNVDPNANPNVD, peptide 29 NANPNANPNANPNAN), SPZ binding (none, low, high), SPZ neutralization (none, very low, low, moderate, high), and references used to classify mAbs. Undet., undetectable; n/a, not applicable. (DOCX) [file ppat.1010133.s001.docx]

| **mAb** | **Isotype** | **Species** | **Antigen** | **BLI Apparent Avidity (nM)** | | | **SPZ Binding** | **SPZ Neutralization** | **Reference** |
| --- | --- | --- | --- | --- | --- | --- | --- | --- | --- |
|  |  |  |  | **Pep21** | **Pep22** | **Pep29** |  |  |  |
| VRC01 | IgG | Human | HIV-1 gp120 | n/a | n/a | n/a | None | None | [1] |
| 5D5 | IgG | Mouse | N-CSP | n/a | n/a | n/a | Low | None | [2] |
| CIS43 | IgG | Human | CSP Repeats  NPDP | <0.001 | 1.5 | undet. | High | High | [3,4] |
| CIS42 | IgG | Human |  | 0.26 | 0.90 | 2.29 | High | Very Low | [3]  This Study |
| L9 | IgG | Human | CSP Repeats  NVDP | 6.0 | 0.28 | undet. | High | High | [4] |
| F10 | IgG | Human |  | 1.50 | 11.32 | undet. | High | Very Low | This Study |
| mAb10 | IgG | Human | CSP Repeats  NANP | 1.6 | 8.2 | <0.001 | High | Moderate | [3,4] |
| MGG4 | IgG | Human |  | 4.28 | 2.72 | <0.001 | High | Low | [5]  This Study |
| MGU12 | IgG | Human |  | 2.1 | 3.9 | <0.001 | High | Low | [4,5] |
| 1210 | IgG | Human |  | 2.2 | 100 | 0.043 | High | Low | [4,6] |
| 311 | IgG | Human |  | 4.1 | 3.0 | <0.001 | High | Moderate | [4,7] |
| 317 | IgG | Human |  | 1.2 | 0.49 | <0.001 | High | High | [4,7] |
| mAb4 | IgG | Human |  | undet. | 7.24 | 0.39 | High | Very Low | [3]  This Study |
| L4 | IgG | Human | C-CSP | n/a | n/a | n/a | Low | None | This Study |
| L7 | IgG | Human |  | n/a | n/a | n/a | Low | None | This Study |
| L15 | IgM | Human |  | n/a | n/a | n/a | Low | None | This Study |
| L20 | IgM | Human |  | n/a | n/a | n/a | Low | None | This Study |
| L37 | IgM | Human |  | n/a | n/a | n/a | Low | None | This Study |
| L40 | IgM | Human |  | n/a | n/a | n/a | Low | None | This Study |
| R1 | IgG | Human |  | n/a | n/a | n/a | Low | None | This Study |
| R2 | IgG | Human |  | n/a | n/a | n/a | Low | None | This Study |
| R3 | IgG | Human |  | n/a | n/a | n/a | Low | None | This Study |
| R4 | IgG | Human |  | n/a | n/a | n/a | Low | None | This Study |
| R7 | IgM | Human |  | n/a | n/a | n/a | Low | None | This Study |
| mAb15 | IgG | Human |  | n/a | n/a | n/a | Low | None | [3] |
| 171­0 | IgG | Human |  | n/a | n/a | n/a | Low | None | [8] |

**References**

1. Zhou T, Georgiev I, Wu X, Yang Z-Y, Dai K, Finzi A, et al. Structural basis for broad and potent neutralization of HIV-1 by antibody VRC01. Science. 2010 Aug 13;329(5993):811–7.

2. Espinosa DA, Gutierrez GM, Rojas-López M, Noe AR, Shi L, Tse S-W, et al. Proteolytic Cleavage of the Plasmodium falciparum Circumsporozoite Protein Is a Target of Protective Antibodies. J Infect Dis. 2015 Oct 1;212(7):1111–9.

3. Kisalu NK, Idris AH, Weidle C, Flores-Garcia Y, Flynn BJ, Sack BK, et al. A human monoclonal antibody prevents malaria infection by targeting a new site of vulnerability on the parasite. Nat Med. 2018 May;24(4):408–16.

4. Wang LT, Pereira LS, Flores-Garcia Y, O’Connor J, Flynn BJ, Schön A, et al. A Potent Anti-Malarial Human Monoclonal Antibody Targets Circumsporozoite Protein Minor Repeats and Neutralizes Sporozoites in the Liver. Immunity. 2020 Oct 13;53(4):733-744.e8.

5. Tan J, Sack BK, Oyen D, Zenklusen I, Piccoli L, Barbieri S, et al. A public antibody lineage that potently inhibits malaria infection through dual binding to the circumsporozoite protein. Nat Med. 2018 May;24(4):401–7.

6. Imkeller K, Scally SW, Bosch A, Martí GP, Costa G, Triller G, et al. Antihomotypic affinity maturation improves human B cell responses against a repetitive epitope. Science. 2018 Jun 22;360(6395):1358–62.

7. Oyen D, Torres JL, Wille-Reece U, Ockenhouse CF, Emerling D, Glanville J, et al. Structural basis for antibody recognition of the NANP repeats in Plasmodium falciparum circumsporozoite protein. Proc Natl Acad Sci USA. 2017 28;114(48):E10438–45.

8. Scally SW, Murugan R, Bosch A, Triller G, Costa G, Mordmüller B, et al. Rare PfCSP C-terminal antibodies induced by live sporozoite vaccination are ineffective against malaria infection. J Exp Med. 2018 02;215(1):63–75.
