# Supplementary figures and images for: Protective effects of combining monoclonal antibodies and vaccines against the Plasmodium falciparum circumsporozoite protein
Source: PLoS Pathog. 2021 Dec 6;17(12):e1010133. doi: 10.1371/journal.ppat.1010133 (PMC8675929; doi:10.1371/journal.ppat.1010133)

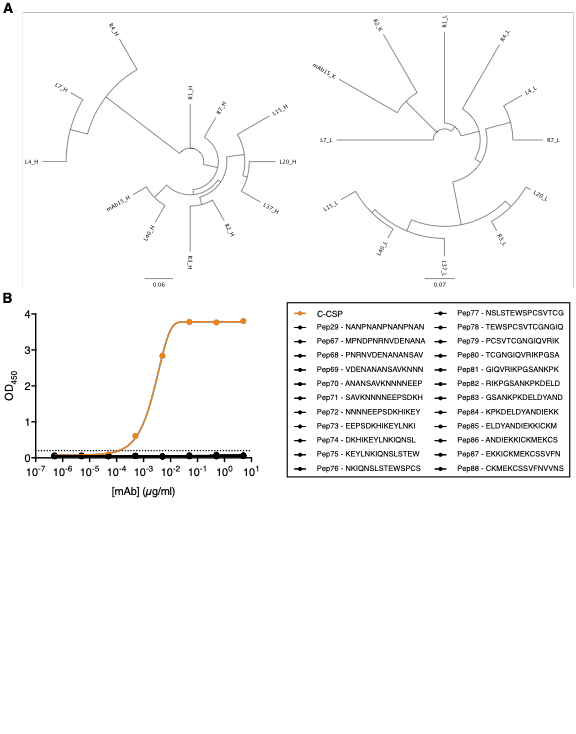

Supplement: S1 Fig — (A) Phylogenetic trees of the heavy chain (left) and light chain (right) sequences of the C-CSP mAbs in this study. 1710 was not included as its original nucleotide sequence could not be retrieved. (B) Binding of varying concentrations of thirteen pooled C-CSP-specific mAbs (L4–1710) to 15mer overlapping peptides numbered 67–88 as determined by ELISA. Optical density at 450 nm (OD450) is plotted; peptide sequences are depicted. C-CSP and the NANP-containing peptide 29 were included as positive and negative controls, respectively. (TIFF) [file ppat.1010133.s004.tiff]

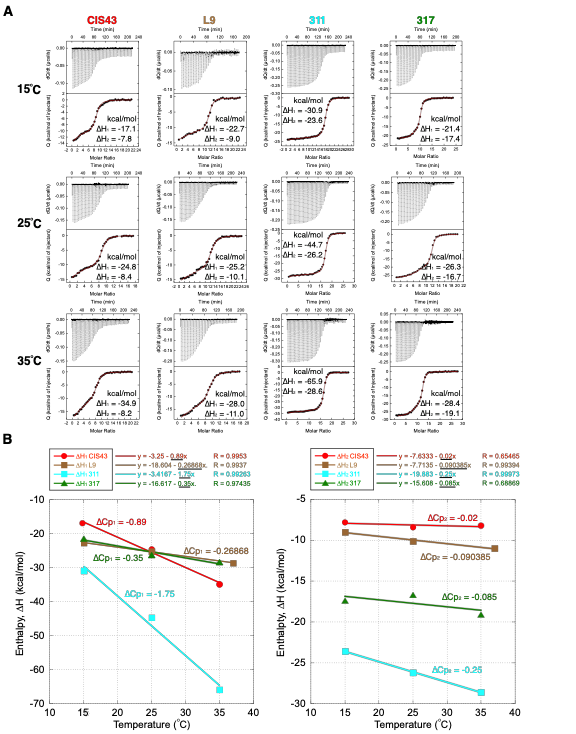

Supplement: S2 Fig — (A) ITC plots of CIS43, L9, 311, and 317 IgG binding to FL-rCSP at indicated temperatures (15–35°C). Top, dQ/dt (change in heat flow, Q, as a function of time, t). Bottom, the integrated heat associated with each IgG injection shown as a function of the molar ratio between IgG antigen binding sites and FL-rCSP in the calorimetric cell. The red line represents the result from best nonlinear least squares fit of the data. Enthalpy values of the first and second binding events (ΔH1 and ΔH2, respectively) are shown. (B) Plots of the enthalpy changes ΔH as a function of temperature (15–35°C) for the binding of each mAb to the first (ΔH1, left panel) and second (ΔH2, right panel) sets of sites, respectively. The equation of each line (y = mx + b) and correlation coefficient (R) are depicted. The change in heat capacity (ΔCp) associated with each binding event is equal to m (the slope of the line) and is underlined in the equation of the line and depicted in the plots for each mAb. (TIFF) [file ppat.1010133.s005.tiff]

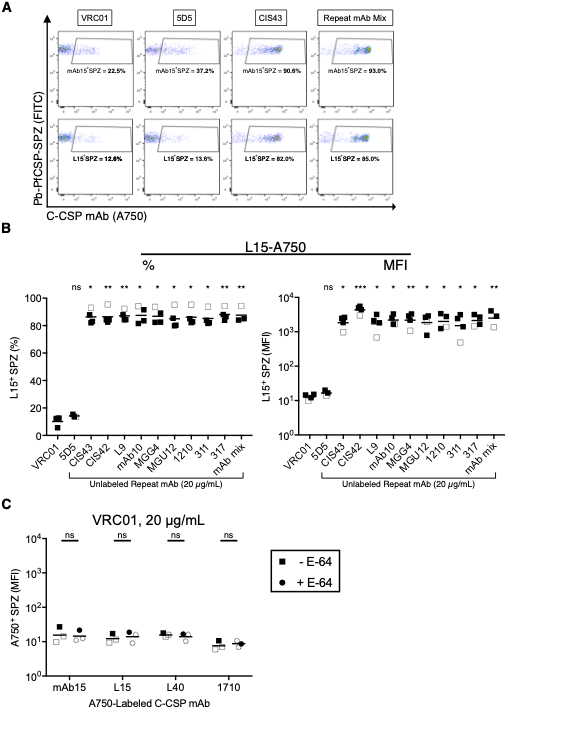

Supplement: S3 Fig — (A) Representative flow cytometry plots depicting 20 μg/mL mAb15-A750 (top panel) or L15-A750 (bottom panel) binding to SG Pb-PfCSP-SPZ in the presence of 20 μg/mL unlabeled VRC01, 5D5, CIS43, and repeat mAb mix (mAb mix, CIS43-317 in B). Percentages of mAb15+ or L15+SPZ are shown. (B) Percentage and MFI of Pb-PfCSP-SPZ (filled squares) and PfSPZ (open squares) bound by 20 μg/mL L15-A750 when co-incubated with 20 μg/mL of specified unlabeled mAb. P-values were determined by comparing PfCSP mAbs to VRC01 using the Kruskal-Wallis test. (C) Binding of four A750-labeled C-CSP mAbs (20 μg/mL) to Pb-PfCSP-SPZ (filled symbols) or PfSPZ (open symbols) when co-incubated with unlabeled VRC01 (20 μg/mL), with or without the protease inhibitor E-64. P-values were determined by comparing -E-64 to +E-64 for each C-CSP mAb using a two-way ANOVA with Sidak’s post-hoc correction. (B, C): ***, p<0.001; **, p<0.01; *, p<0.05; ns (not significant), p>0.05. (TIFF) [file ppat.1010133.s006.tiff]

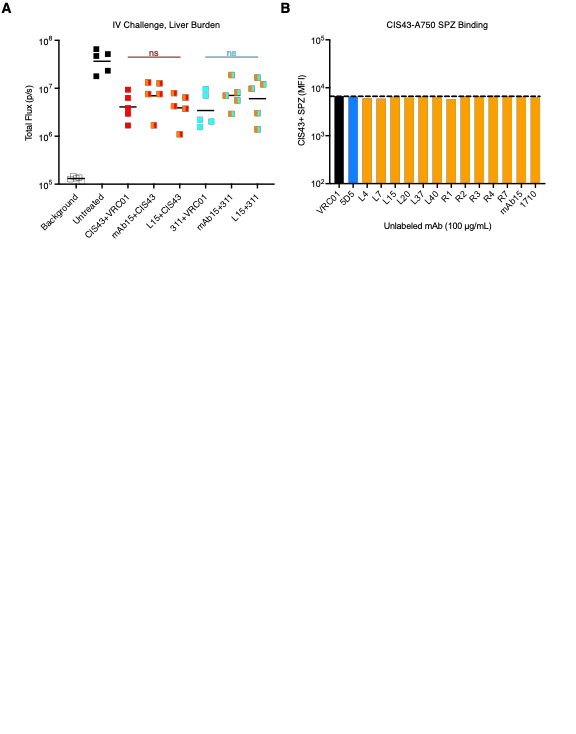

Supplement: S4 Fig — (A) Liver burden in mice (n = 5/group; line indicates geometric mean) 40 hours post-challenge mediated by indicated mAb combinations (CIS43 and 311, 50 μg; VRC01, mAb15, L15, 300 μg) administered 2 hours before IV challenge with 2,000 Pb-PfCSP-SPZ. P-values were determined by comparing repeat mAb + VRC01 to untreated control (black values) or C-CSP mAb + repeat mAb to repeat mAb + VRC01 (colored values) using the Kruskal-Wallis test with Dunn’s post-hoc correction. *, p<0.05; ns (not significant), p>0.05. (B) MFI of Pb-PfCSP-SPZ bound by 2 μg/mL CIS43-A750 when co-incubated with 100 μg/mL of specified unlabeled mAb. (TIFF) [file ppat.1010133.s007.tiff]

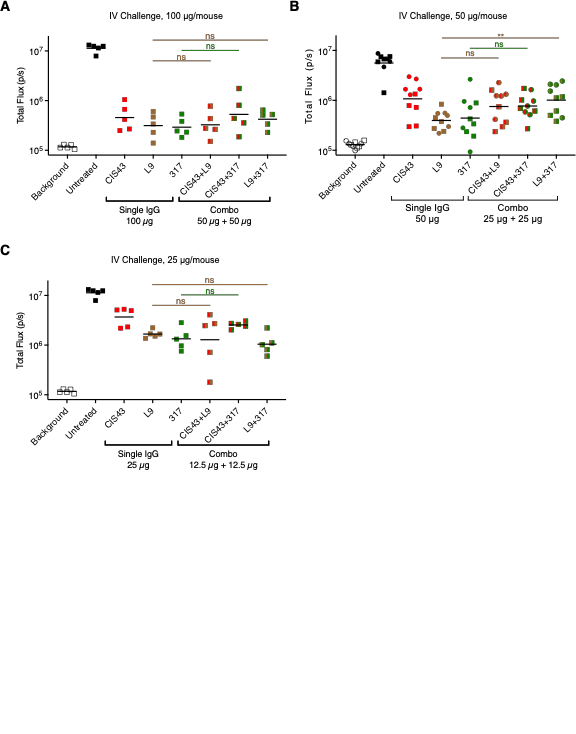

Supplement: S5 Fig — (A-C) Liver burden 40 hours after IV challenge with 2,000 Pb-PfCSP-SPZ in mice (n = 5-10/group; 50 μg data in E was combined from two experiments, circles and squares) that received CIS43, L9, and 317 alone (A, 100 μg; B, 50 μg; C, 25 μg) or in combination (A, 50+50 μg; B, 25+25 μg; C, 12.5+12.5 μg). Black lines represent geometric mean. P-values were determined by comparing mAb combinations to L9 or 317 alone using the two-tailed Mann-Whitney test. ***, p<0.001; **, p<0.01; *, p<0.05; ns (not significant), p>0.05. (TIFF) [file ppat.1010133.s008.tiff]

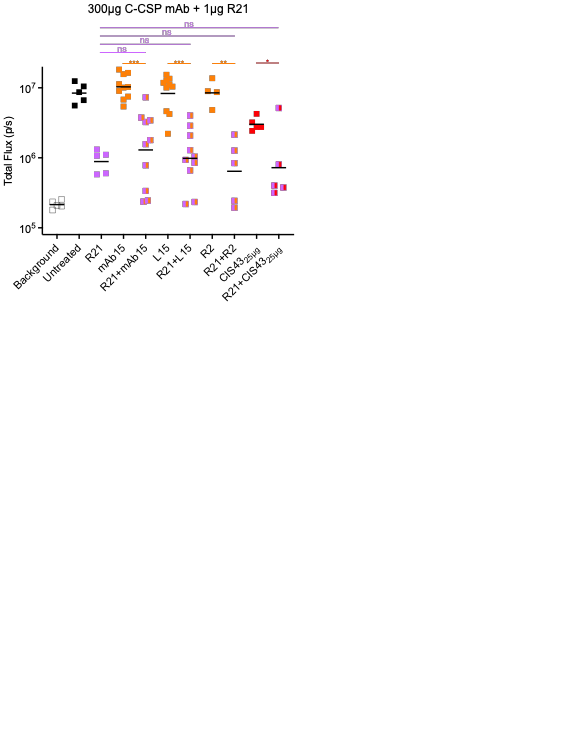

Supplement: S6 Fig — Liver burden 40 hours after IV challenge with 2,000 Pb-PfCSP-SPZ in mice (n = 5-10/group) immunized with 1 μg R21 alone; mice administered 300 μg of C-CSP mAbs (mAb15, L15, R2) or 25 μg of CIS43 alone; and mice immunized with 1 μg R21 and administered 300 μg of C-CSP mAbs (mAb15, L15, R2) or 25 μg of CIS43. P-values were determined by comparing each R21+mAb combination to the R21 alone or respective mAb alone groups using the Kruskal-Wallis test. ***, p<0.001; **, p<0.01; *, p<0.05; ns (not significant), p>0.05. (TIFF) [file ppat.1010133.s009.tiff]
