# Supplementary material for: Protective effects of combining monoclonal antibodies and vaccines against the Plasmodium falciparum circumsporozoite protein
Source: PLoS Pathog. 2021 Dec 6;17(12):e1010133. doi: 10.1371/journal.ppat.1010133 (PMC8675929; doi:10.1371/journal.ppat.1010133)
Supplement: Supplementary file 3 [file ppat.1010133.s002.docx]

mAb	V_H	% SHM	V_H Amino Acid Sequence	V_L	% SHM	V_L Amino Acid Sequence
L4	VH4-39*01	5.5	QLQLQESGPRLVKPSETLSLTCTVSGGSISRTSYYWDWIRQPPGKGLEWIGSIYYRGSTYHNPSLKSRVTISVDTSKNQFSLRLTSVTAADTAVYYCARPKNLGVVAATNWFDPWGQGTLVTVSS	Vλ1-40*01	1.74	QSVLTQPPSVSGAPGQRVTISCTGSSSNIGATYDVHWYQQLPGTAPKLLIYDNNNRPSGVPDRFSGSKSGTSASLAITGLQADDEADYYCQSYDSSLSGVVFGGGTKLTVL
L7	VH4-39*01	2.06	QLQLQESGPGLVKPSETLSLTCTVSGGSISSSNYYWDWIRQPPGKGLEWIGSINYSGSTYYNPSLKSRVTISVDTSKIQFSLKMSSVTAADTAVYYCARRRVDFWSGYNWFDPWGQGTLVTVSS	Vλ9-49*01	1.35	QPVLTQPPSASASMGASVTLTCTLSSGYSKYKVDWYQQRPGKGPRFVMRAGTGGIVGSKGDGIPDRFSVLGSGLNRYLTIKNIQEEDESDYHCGADHGSGSNFVYVFGTGTKVTIL
L15	VH3-43*01	1.04	EVQLVESGGVVVQPGGSLRLSCAASGFTFDDYTMHWVRQAPGKGLEWVSLISWDGGTTYYADSVKGRFTISRDNSKNSLYLRMNSLRTEDTALYYCAKGLVAQWLRGDYYYGLHVWGQGTTVTVSS	Vλ2-14*03	1.74	QSALTQPASVSGSPGQSITISCTGTSSDVGAYNSVSWYQQHPGKAPKLMIYDVTNRPSGVSNRFSGSKSGNTASLTISGLQAEDEADYYCSSYTITNTLVFGGGTKLTVL
L20	VH3-9*01	1.39	EVQLVESGGGLVQPGRSLRLSCAASGFTFDDYAMHWVRQAPGKGLEWVSGINWNSGSIGYADSVKGRFTISRDNARNSLYLQMNGLRAEDTAFYYCAKDLRAYYYGSGSPWGFHHWGQGTLVTVSS	Vλ2-8*01	1.04	QSALTQPPSASGSPGQSVTISCTGTSSDVGGYNYVSWYEQHPGKAPKLLIYEVSKRPSGVPDRFSGSKSGNTASLTVSGLQAEDEADYYCSSYAGSNNVVFGGGTKLTVL
L37	VH3-9*01	1.26	EVQLVESGGGLVQPGRSLRLSCAASGFTFDDYAMHWVRQAPGKGLEWVSGISWNSGSIGYAVSVKGRFTISRDNAKNSLYLQMNSLRAEDTALYYCAKDIKAQWLTDGLLFYYGLNVWGLGTTVTVSS	Vλ2-23*01	1.04	QSALTQPASVSGSPGQSITISCTGSTSDVGSYNLVSWYQQHPGKAPRLMIYEGSKRPSGVSNRFSGSKSGNTASLTISGLQAEDEADYYCCSYAGSSTLVFGAGTKVTVL
L40	VH3-30*03	3.82	EVQLVESGGGVVQPGRSLRLSCAASGFTFNNYGMHWVRQAPGKGLEWVAVISYEGRNKNYADSVKGRFTISRDSSKNTVYLQMNSLRPEDTAVYYCAKDRHAPIWSGPVRYYYYGMDVWGQGTTVTVSS	Vλ2-14*01	0.69	QSALTQPASVSGSPGQSITISCTGTSSDVGGYNYVSWYQQQTGKAPKLMIYEVSNRPSGVSNRFSGSKSGNTASLTISGLQAEDEADYYCSSYTSSSTPYVFGTGTKVTVL
R1	VH3-15*01	1.02	EVQLVESGGGLVKPGGSLRLSCAASGFIFTNAWMSWVRQAPGKGLEWVGRIKSKTDGGTTDYAAPVKGRFTISRDDSKKTLYLQMNSLKTEDTAVYYCTRDPRVEIGPFDYWGQGTLVTVSS	Vλ7-43*01	1.39	QTVVTQEPSLTVSPGGTVTLTCASSTGAVTSGNYPNWFQRKPGQAPRALIYSTSNKHSWTPARFSGSLPGGKAALTLSGVQPEDEAEYYCLVYYGGARVFGGGTKLTVL
R2	VH3-48*03	0.69	EVQLVESGGGLVQPGGSLRLSCAASGFTFSSYEMNWVRQAPGKGLEWVSYISSSGSTIYYADSVKGRFTISRDNAKNSLYLQMNSLRAEDTAVYYCARDSGRYKDLYYGMDVWGLGTTVTVSS	Vκ1D-12*01	3.23	DIQMTQSPSSVSASVGDRVTITCRASQGISSWLAWYQQKPGKAPKLLIYAASRLQSAGPSRFSGSGSGTDFTLTISSLQPEDFATYYCQQANSFSTFGGGTKVEIK
R3	VH3-11*01	4.17	QMQLVESGGGLVKPGGSLRLSCAASGFTFSDYFMGWIRQAPGKGLEWLSYISYSGSTIYYADSVKGRFTISRDNAKKSLSLQMNGLRVEDTAVYYCAKGRGSYFFGHYYAFDIWGQGTMVTVSS	Vλ2-8*01	2.78	QSALTQPPSASGSPGQSVTISCTGTGSDVGGFNSVSWYQQHPGKAPKLLIYEVSKRPSGVPDRFSGSKSGNTASLTVSGLQAEDEADYYCSSYAGSNIHWVFGGGTKLTVL
R4	VH4-4*02	3.47	QVQLQESGPGLVKPSGTLSLTCAVSGGSIRGGNWWSWVRQPPGKGLEWIGEIYHSGTTNYNPSLKSRVTISVDKSKSQFSLKLSSVTAADTAVYYCARSSFGTIFGEVPFSGFDYWGQGTLVTVSS	Vλ3-25*03	1.79	SYELTQPPSVSVSPGQTARITCSGDALPKQYAYWYQQKPGQAPVMVIYKDSERPSGIPERFSGSSSGTTVTLTISGVQAEDEADYYCQSADSSGSYPYVIFGGGTKLTVL
R7	VH3-23*01	1.04	EVQLLESGGGLVQPGGSLRLSCAASGFTFSSYAMSWVRQAPGKGLEWVSGITSSGGSTYYADSVKGRFTISRDNSKNTLYLQMNSLRAEDTAVYYCAKVGYTSGWPEDYWGQGTLVTVSS	Vλ1-47*02	0.7	QSVLTQPPSASGTPGQRVTISCSGSSSNIGSNYVYWYQQLPGTAPKLLIYINNQRPSGVPDRFSGSKSGTSASLAISGLRSEDEADYYCAAWDDSLSGRVFGGGTKLTVL
mAb15	VH3-33*01	0.69	EVQLVESGGGVVQPGRSLRLSCAASGFTFNSYGMHWVRQAPGKGLEWVAVIWYDGSNKYYADSVKGRFTISRDNSKNTLYLQMNSLRAEDTAVYYCARSRAYDSSGYYYFYYYYGMDVWGQGTTVTVSS	Vκ3-20*01	0.35	EIVLTQSPGTLSLSPGERATLSCRASQSVSSSYLAWYQQKPGQAPRLLIYGASSRATGIPDRFSGSGSGTDFTLTISRLEPEDFAVYYCQHYGSSSWTFGQGTKVEIK
1710	VH3-21	-	EVQLQESGGGLVKPGGSLRLSCAASGFTFSSYSMNWVRQAPGKGLEWVSSITSSSSYIYYADSVKGRFTISRDNAKNSLYLQMNSLRAEDTAVYYCARDPGIAAADNHWFDPWGQGTLVTVSS	Vλ3-1	-	AYELTQPPSVSVSPGQTASITCSGDKLGDKYACWYQQKPGQSPVVVIYQDTKRPSGIPERFSGSNSGNTATLTISGTQAMDEADYYCQAWDSSTVVFGGGTKLTVL
