# Supplementary material for: Protective effects of combining monoclonal antibodies and vaccines against the Plasmodium falciparum circumsporozoite protein
Source: PLoS Pathog. 2021 Dec 6;17(12):e1010133. doi: 10.1371/journal.ppat.1010133 (PMC8675929; doi:10.1371/journal.ppat.1010133)
Supplement: Supplementary file 4 [file ppat.1010133.s003.docx]

	L4	L37	R3	R4	1710	mAb15	L40	R7	R2	mAb10	5D5
L4	[illegible]	4.72	11.16	7.17	9.27	5.32	4.18	1.41	8.48	70.70	5.45
L37	-0.05	[illegible]	-0.14	-0.16	-0.12	-0.09	-0.06	-0.06	2.80	14.04	2.78
R3	-0.16	-0.22	[illegible]	-0.36	-0.24	-0.13	-0.11	-0.07	3.31	29.62	3.01
R4	-0.01	0.03	0.08	[illegible]	-0.04	-0.03	-0.02	0.04	2.96	30.71	2.85
1710	-0.02	-0.05	-0.09	-0.11	[illegible]	-0.07	-0.05	-0.06	3.16	13.32	2.73
mAb15	0.43	0.35	0.70	-0.10	-0.04	[illegible]	-0.02	0.73	2.90	27.00	2.66
L40	1.07	3.30	8.99	5.28	3.11	2.28	[illegible]	0.54	10.74	84.12	4.13
R7	0.25	3.76	5.22	4.22	0.71	4.79	0.06	[illegible]	0.09	47.51	3.82
R2	-0.14	4.05	4.02	2.66	3.38	0.56	3.14	-0.07	[illegible]	23.59	3.14
5D5	4.66	7.19	8.57	4.57	6.33	4.37	5.64	1.59	6.68	40.84	[illegible]
