# Supplementary material for: Protective effects of combining monoclonal antibodies and vaccines against the Plasmodium falciparum circumsporozoite protein
Source: PLoS Pathog. 2021 Dec 6;17(12):e1010133. doi: 10.1371/journal.ppat.1010133 (PMC8675929; doi:10.1371/journal.ppat.1010133)
Supplement: Attachment — Submitted filename: 211023 Response to Reviewers.docx [file ppat.1010133.s010.docx]

**Part I - Summary**
Please use this section to discuss strengths/weaknesses of study, novelty/significance, general execution and scholarship.

Reviewer #1:

Wang et al. report on the in vitro sporozoite recognition and in vivo protection capacities of thirteen monoclonal antibodies (mAbs)that recognize conformational epitopes in the C-terminal domain of PfCSP (C-CSP) with or without combination with mAbs against three tetrapeptides in the PfCSP repeat region. Interestingly, they show that while the combination enhances sporozoite recognition in vitro, it does not enhance protection against challenge in a rodent model of infection. Also of note, the data reported here substantiate the existence of distinct PfCSP conformations on midgut versus salivary gland sporozoites and indicate that C-CSP mAbs are able to distinguish between the two native PfCSP conformations. Additionally, the authors show that mAbs against the repeat region of PfCSP potentiate C-CSP mAb binding to sporozoites by changing the protein’s conformation, albeit without this resulting in an increase in protection in vivo. Finally, they show that mAbs against the repeat region of PfCSP do not synergize in vivo and, unlike mAbs against the N- or C-termini of PfCSP, antagonize the binding of other repeat mAbs to sporozoites. These results are novel and relevant to the field, as they may not only shed light on PfCSP antibody-mediated protection against Plasmodium infection, but also inform on the design on vaccines against malaria. The study is well performed and the conclusions are warranted by the results. Overall this is a study worthy of publication and only a suggestion is made for its improvement.

- **We thank the reviewer for their thoughtful summary of our study.**

Reviewer #2:

This study by the Seder group evaluates the sporozoite binding and antiparasitic capacity of a large panel of human mAbs against different regions of P. falciparum CSP (central repeat and C-terminus) in a mouse model, and report no evidence for C-terminus antibodies, with weak binding alone that is potentiated when combined with anti-repeat mAbs. C-terminus mAbs had no neutralizing effect against sporozoite challenge in passive transfer murine experiments, alone or in combination, in contrast with previously well characterized mAbs against the repeat epitopes. Furthermore, no evidence is found for additive or synergistic neutralizing effect of combination of mAbs in contrast with other diseases, in fact they found that some anti-repeat mAbs (but not anti-C-terminus) could antagonize the effect of other anti-repeat mAbs. The considerations about binding of anti-C-terminus mAbs depending on the open vs closed conformation of the CSP, and the two-step model, are interesting and provide insight into availability (unmasking) of epitopes for proper function. The strength and novelty relies on the detailed analysis of a panel of mAbs from humans immunized with sporozoite vaccines. The main limitation is the strong focus on the neutralization function that is not a correlate of protection in humans and the limitations of the transgenic mouse model used that may not reflect the in vivo biological processes in African individuals upon exposure and vaccination. As a consequence, a role for CSP C-terminus Abs and an enhanced protection in combination with anti-repeat Abs via other mechanisms still cannot be discarded. This could be reflected better in the title (that could include the term neutralization), abstract and discussion/conclusions.

- **We thank the reviewer for their thoughtful summary of our study, including its strengths and limitations.**

Reviewer #3:

Yang et al present a dissection of the in vitro and in vivo activities of a panel of monoclonal antibodies to the C-terminus of PfCSP, and also investigate potential for beneficial interactions between combinations of CSP-repeat-specific mAbs in in vivo protection. The study attempts to shed light upon an uncertainty about the role of C-CSP-specific antibody in protection induced by the RTS,S malaria vaccine.

The key positive finding is that antibodies to CSP repeat result in increased accessibility of the CSP C-terminus for binding of these mAbs. The remaining results are essentially negative: in particular, the authors do not find evidence of a protective effect of the C-CSP-specific antibodies, with or without the addition of CSP-repeat-specific antibodies.

Negative results can of course be very important, but in this case it is probably not possible to say whether the absence of a protective effect is due to a characteristic of these specific mAbs / these specific in vivo & in vitro assays, or whether C-CSP-specific antibodies in general lack the potential for protection. In some ways mAbs may not be the ideal tool to resolve the uncertainty about the role of C-CSP-specific antibody: it is difficult to know whether a mAb panel is representative of the range of activities which may be achieved by polyclonal antibody. Use of antigen-affinity-purified C-CSP-specific polyclonal antibody as a complementary reagent in some of the work might have been helpful. The authors neither replicate nor attempt to refute the conflicting previous observation that C-CSP-specific polyclonal Ab can inhibit sporozoite invasion, and the authors do not investigate the ability of the novel mAbs to trigger other mechanisms like phagocytosis and complement fixation, which the introduction highlights have been suggested to be involved in protection.

I think ability to draw generalizable conclusions from this work about mechanisms of vaccine-induced production will therefore be limited, unless the authors are able to more fully compare the effects of these mAbs with those of vaccine-induced C-CSP-specific pAb using a more comprehensive set of assays. I think the main contribution of this article might instead be to make available a set of new reagents to the community (although as far as I can see it is not clear whether/how the mAbs will be available). Further work is needed to clarify how these new antibodies are differentiated from each other and from previously reported mAbs (major point 1 below), and perhaps to move the emphasis of the manuscript away from the negative results. Although the conclusions are worded carefully and appropriately, the manuscript reads a little like an unsuccessful effort to find a protective effect, rather than a considered effort either to disprove the possibility of a protective effect or to fully characterise a new set of reagents.

- **We thank the reviewer for their thoughtful summary of our study, including its strengths and limitations.**

**Part II – Major Issues: Key Experiments Required for Acceptance**
Please use this section to detail the key new experiments or modifications of existing experiments that should be absolutely required to validate study conclusions.

Generally, there should be no more than 3 such required experiments or major modifications for a "Major Revision" recommendation. If more than 3 experiments are necessary to validate the study conclusions, then you are encouraged to recommend "Reject".

Reviewer #1: None

Reviewer #2:

The major issue is the framework in which the study is presented, ie. the adequacy of the model and assumptions for generalizability of results. The in vivo protection against sporozoite challenge in mice and redout chosen may not reflect the mechanisms of in vivo protection in humans mediated by those antibodies.

- Neutralizing Abs are not a correlate of protection therefore it is possible that other mechanisms are more relevant for the tested mAbs

- **We have added additional references in the Introduction and Discussion to more clearly acknowledge that direct binding and neutralization by CSP antibodies (i.e., antibodies reducing sporozoite motility and hepatocyte invasion by binding CSP, which is required for both of these phenotypes) may not be the only mechanisms by which CSP antibodies prevent sporozoites from infecting hepatocytes. Specifically, we state that Fc effector functions (e.g., phagocytosis, complement activation) may contribute to protection and more clearly acknowledge that the *in vivo* mouse model employed in our study may not fully recapitulate the biology of humans infected with PfSPZ and that further *in vitro* studies with human cell lines (THP-1 monocyte lines, HC-04 hepatocyte lines) and PfSPZ might be done to extend our *in vivo* studies.**

- Need to refer to other articles having evidence in the direction that combination of CSP repeat + C-terminus Abs having a more potent role than individually with different readouts

- **We have cited additional references and inserted new sentences in the Introduction and Discussion, which acknowledge that repeat + C-CSP mAb combinations might potentially be more protective than each mAb class alone if tested with different readouts (e.g., *in vitro* with human cell lines or *in vivo* with CHMI). It should be noted that there are no published studies assessing whether combinations of repeat + C-CSP mAbs provide enhanced *in vivo* protection against SPZ challenge in mice compared to each type of mAb alone, and that our study is the first to answer this question.**

Reviewer #3:

1. The authors should present data to make clear whether the various C-CSP-specific mAbs are truly distinct from each other, or from the previously-published C-CSP-specific mAbs, in terms of clonal relatedness and binding competition. Sequence analysis and a binding competition assay should be relatively straightforward. Ideally, as one of the arguments for publication of this article in PLoS Pathogens rather than a more specialist journal is that it reports a panel of new reagents which may be of use to the community, the amino acid sequences of the new mAbs should be reported so that they can be produced and used by others.

- **In terms of C-CSP mAb sequence analysis, we have added phylogenetic trees (S1A Fig) showing how clonally related the C-CSP mAb VH/VL sequences are and Supplemental Table 2 detailing the gene families, somatic mutation percentages, and amino acid sequences of the C-CSP mAb VH/VL used in this study.**
- **In terms of C-CSP mAb binding competition assays, we used surface plasmon resonance (SPR) on the Carterra LSA platform to perform epitope binning of the C-CSP mAbs (Fig 2B). Note that we have replaced the BLI data with the C-CSP epitope binning SPR data, as we feel this data is more informative as it provides insight into where the C-CSP mAbs bind in FL-rCSP relative to each other.**

2. (Modification of analysis rather than additional experiment) There is a lack of clarity about what synergism, additivity and sub-additivity really mean with respect to the action of combinations of monoclonals, and hence a lack of clarity about how to test for them (or the extent to which the unavoidable use of a complex in vivo assay restricts the feasibility of testing for them). Stemming from this, I think the conclusions / figure titles / data analysis regarding the in vivo effects of mAb combinations (Figures 3, 4, S5) should be revised.

At present there are confusing webs of stars & lines on Figures 4 and S5 (and to a lesser extent Figure 3), indicating multiple statistical comparisons on the figures looking at the in vivo effects of pairs of antibodies. In each case more clarity is needed on what the key hypothesis was, how it was tested, and how this should be interpreted.

Comparison to the untreated control isn’t really very relevant – with the exception of the non-neutralising anti-repeat mAbs in Figure 4C, it’s clear that all tested mAb treatments are protective. Instead I think the authors are trying to determine, by looking for differences between certain pairs of treatments, what can be said about whether any such differences (or the lack of them) are indicative of sub-additive, additive (i.e. independent) or synergistic interactions between the treatments.

The basic design of the in vivo experiments (comparing ‘X ug of mAb A + X ug of mAb B’ vs ’2X of A’ vs ‘2X of B’) is not really sufficient to detect anything other than a major synergistic effect (for which the effect of A+B would be better than seen with either 2A or 2B). Such synergy is not seen.

Without groups receiving X ug of A alone (not 2X), and X ug of B alone (or ideally dose response curves for X and Y alone), and some further maths to identify the expected result if A & B were acting independently, I don’t think it is possible to distinguish between weak synergy, additivity, or sub-additive (interfering) activities. I think all the data presented could be consistent with an additive effect- the combinations of half-doses of two mAbs don’t appear worse than full doses of a single mAb.

I would not advocate re-performing the experiments with additional groups, but the analysis and interpretation should be reviewed and made more precise.

- **We agree that further *in vivo* studies suggested by the reviewer (e.g., dose response curves) and additional mathematical analyses would have enabled clearer definition of interactions between the mAbs (weak synergy, additivity, sub-additivity) and thank the reviewer for providing suggestions to re-interpret our analyses. Accordingly, we have removed all characterizations of *in vivo* protection results as “additive” or “synergistic” and replaced them with more general terms such as “cooperative neutralization” or “improved protection.”**
- **We have performed a new study, shown in Fig 6A, where we included the additional controls suggested by the reviewer (i.e., X µg of A alone and X µg of B alone). Specifically, this study compares CIS43, L9, or 317 alone (50 µg/mouse) to combinations of CIS43, L9, and 317 (CIS43+L9, CIS43+317, L9+317; 25+25 µg) and combinations of CIS43, L9, and 317 with isotype control VRC01 (CIS43+VRC01, CIS43+VRC01, L9+VRC01; 25+25 µg). The results of this study reinforce our conclusion that combinations of two potent PfCSP repeat mAb do not provide improved protection compared to single PfCSP repeat mAbs.**
- **We have simplified the statistical comparisons for the *in vivo* protection figures so that the statistical comparisons are only against the relevant single mAb controls. For the C-term + repeat mAb protection figures (Fig 5 and S4 Fig), we compare C-term + repeat mAb combinations to the repeat mAb alone (either mAb10, CIS43, or 311). For the repeat mAb combination figures (Fig 6 and S5 Fig), we compare each repeat mAb combination to the most potent repeat mAb in the combination (CIS43+L9 vs. L9 | CIS43+317 vs. 317 | L9+317 vs. L9). For the new data in Fig 6A, which had the PfCSP mAb + VRC01 control, we also included an analysis where we compared the PfCSP mAb combination (e.g., CIS43+L9, 25+25 µg) to its respective CIS43+VRC01 and L9+VRC01 (25+25 µg) controls.**

**Part III – Minor Issues: Editorial and Data Presentation Modifications**
Please use this section for editorial suggestions as well as relatively minor modifications of existing data that would enhance clarity.

Reviewer #1:

The paper is well organized, the data are clearly presented, and the results are adequately shown and discussed. However, I would suggest that the authors consider moving some of the data currently presented as Supplementary information to the main figures of the article. Specifically, I would say that the Scheme at the top of Fig. S1-A is informative enough to be shown in the main article, and that the data in Fig. S2 (or a selection thereof) and Fig. S5 are important enough to warrant their inclusion as main figures in the manuscript.

- **We have moved Fig S1A and Fig S2 into the main figures as Fig 1 and Fig 3. While we agree that Fig S5 would ideally be included in Fig 6, its inclusion would make Fig 6 less clear. Fig 6 is currently organized to show the 100 µg protection data next to the *in vitro* sporozoite binding data for both the potent repeat mAbs (CIS43, L9, 317) and the non-potent mAbs (CIS42, F10, mAb4). We feel that this way of presenting the data is important to highlight the lack of improved protection and the antagonism of mAb binding (i.e., the main idea of the figure) and that including the extra data from Fig S5 (which only confirms the data in Fig 6) is less important than maintaining this symmetry.**

Reviewer #2:

Abstract
- Indicate the source of the human mAbs

- **A description of the source of the human mAbs has been added to the Abstract**

Introduction
- Acknowledge that there may be other non-neutralizing functions of sporozoite antibodies and provide references

- **We have noted (with references) that, in addition to direct neutralization by blocking sporozoite motility or invasion of hepatocytes, CSP antibodies can also mediate protection by Fc-mediated effector functions such as opsonophagocytosis or complement fixation.**

- In addition, CSP binding (titers) correlate with neutralizing activity, however this is not a surrogate of protection and thus other functions in addition to neutralization should be considered, not only in the RTS,S context but also in naturally acquired and attenuated sporozoite immunity

- **We edited the text to highlight the Suscovich et al. study, which performed systems serology analysis of serum from RTS,S vaccinees and found that opsonophagocytosis and engagement of Fc gamma receptor 3A were correlated with RTS,S protection. We also highlighted the studies by Kurtovic et al. and Zenklusen et al., which respectively showed that naturally-acquired CSP antibodies fix complement and that immunization with attenuated sporozoites in humans induced complement-fixing antibodies.**

- Make a more clear distinction in the literature refered as to what are human studies and what are mice studies, which vaccine, and whether tested in naive or exposed people, in adults or children.

- **We have made changes to define the species, vaccines, exposure status, and age of previous studies cited more clearly in our manuscript.**

- Review the completeness and adequacy of some key references, for example Ref 5 is a small study about booster responses to CSP but a prior larger study after primary vaccination including anti-repeat and anti-C-terminus CSP Abs is not mentioned, and is required to complement Ref 6 that only assessed anti-repeat IgG titers. Ref 18 reports association IgG2 with protection while other studies not refered report the opposite.

- **We have added additional references of the recent NEJM and Lancet papers showing the results of the RTS,S/AS01 Phase 3 trial to substantiate the data in the Sanchez et al. (formerly ref 5) and White et al. (formerly ref 6) papers. We have inserted two additional references to accompany Suscovich et al. (formerly ref 18) that reported the IgA2 and IgG2 correlation with protection. Specifically, we inserted Chaudury et al. showing that IgG4 is correlated with protection and Ubillos et al. showing that IgG2/4 are correlated with risk while IgG1/3 are correlated with protection.**

Results
-C-CSP-specific mAbs had limited binding to sporozoites (as said in abstract) or no binding (as said in results)?

- **We do not see where in the Results section we say, “no binding.” We do note that the C-CSP mAbs showed “low *in vitro* binding to sporozoites.”**

Discussion
- There is no reference about the potential relevance of glycosilation on the C-terminus, since it was expressed in mammalian cells this may be warranted

- **We have added a sentence acknowledging the potential relevance of CSP/C-term glycosylation on mAb binding, and inserted the Scally et al. reference which showed that mammalian-expressed FL-rCSP did not affect binding of the C-CSP mAb 1710, which we included in our panel.**

- In general, even though the authors refer to field studies of malaria vaccines showing different results with polyclonal responses to C-terminus, it would be necessary to more clearly acknowledge the fact that the lack of consistency between their data and the field trials data could indicate limitations in their readouts and model, as mentioned above (particularly the sole focus on neutralization activity by mAbs)

- **We have edited a paragraph in the Discussion to more clearly acknowledge the limitations of the protection readouts and models used in our study and how these might explain the lack of consistency with the RTS,S serum protection correlation studies. We would like to note that it is unclear what the reviewer is referring to when they say, “neutralization function that is not a correlate of protection in humans.” Do they mean direct binding/neutralization (i.e., inhibition of sporozoite motility, cytotoxicity, CSP reaction, inhibition of hepatocyte traversal/invasion) or Fc-mediated effector functions like phagocytosis and complement fixation? The advantage of testing these mAbs *in vivo* in mice is that all of these mechanisms (i.e., direct antibody neutralization or antibody-dependent effector functions) can potentially contribute to protection, whereas assessment *in vitro* only recapitulates some of these mechanisms.**

Reviewer #3:

1. Given the large number of mAbs used in the study, and the non-intuitive nomenclature, it is not easy for the reader to follow what epitopes are bound by whichever mAb is under discussion at a given point. Could the authors devise some more helpful nomenclature / ‘coding’ system – e.g. use, across all figures, consistent colour-coding of mAbs by binding specificity?

- **To aid the ability of readers to visualize where the PfCSP mAbs used in our study bind, we have moved the graphic of PfCSP that was formerly Fig S1A (detailing the approximate epitopes of all the mAbs in the study) into the main figures as Fig 1. We have also color-coded the mAbs. All C-CSP mAbs are now orange. The main repeat mAbs described (mAb10, CIS43, L9, 317) are respectively colored purple, red, brown, and green. Please also note that we moved the former Fig S2 (showing the comparison of C-CSP mAb binding to SG- vs. MG-SPZ) into the main figures as Fig 3 and changed the plot type for Fig 3B so that it would match the color scheme of the SG- and MG-SPZ in Fig 3A (data is still the same, just different representation).**

2. Line 33-34 ‘did not provide enhanced protection’ requires statement of what the lack of enhancement was in comparison with (presumably C-CSP mAbs alone).

- **We have added a phrase clarifying that the comparison was to C-CSP mAbs alone.**

3. The author summary is not very clearly written, especially lines 49-53 – it could benefit from being re-worded.

- **We have re-worded the specified lines in the Author Summary to improve clarity.**

4. Fig 1A: An AUC heat map is not a particularly common way of reporting ELISA data. Please provide further explanation of how this was produced.

- **The goal of Fig 1A (now Fig 2A) was to provide a gross classification of the 13 C-CSP mAbs and selected controls based on their binding to the three domains of PfCSP (N-CSP, repeats, and C-CSP), not to make any statements about their relative binding affinities measured by ELISA. The heat map clearly demonstrates that the 13 C-CSP mAbs only bind to FL-rCSP and C-CSP. We feel that the AUC heat map was an appropriate illustration to achieve this goal; furthermore, we also note that a previous study describing human C-CSP mAbs (Scally et al, JEM, 2017) also reported their ELISA data as an AUC heat map. We have added further details of how the heat map was generated in the Methods, under the ELISA sub-section.**

5. Fig 1B: BLI data does not all appear to be well fitted by the 1:1 model (it is not explained why the red ‘model-fit’ lines are not shown for all concentrations). Ability to interpret the kinetics is compromised because, with IgG in solution, the binding will not be 1:1. Could this assay not have been run either using Fabs or with the CSP in solution, so true affinity could be measured from a true 1:1 interaction?

- **We have removed the BLI data as it did not provide any additional insight into the main idea of the paper (i.e., combining repeat- and C-CSP-specific mAbs to improve SPZ binding and neutralization). We have instead replaced the BLI data with the epitope binning generated by binding competition assays using surface plasmon resonance.**

6. Fonts on some figures are very small indeed & it would be helpful to consider rearranging / redesigning the figures to improve legibility – eg fig 2, supp fig 1A. Similarly, some panels try to represent quite complex data sets and become quite confusing even though the key result could be shown more simply, perhaps by moving some of the information to supplementary (e.g. the gating shown in 2B could be supplementary or reduced to a single example, and the transgenic spz data sets on panels 2C & 2E are superfluous- the wt Pf data makes the point).

- **We have resized the fonts and redesigned figure panels to make them clearer, and have moved some data to the Supplemental Information.**

7. Figure S3 & associated description in results section: this biophysics data, and the conclusions drawn from it, are quite complex. I suspect many/most readers would require further explanation to grasp what is being shown. I am unable to comment on whether the data shown supports the conclusions being drawn.

- **We have made changes to Fig S3 (now Fig S2) and its legend highlighting the most important mathematical factor calculated from the ITC data, ∆Cp (change in heat capacity). ∆Cp is equal to the slope, m, of the equation of the line (y = mx +b) depicted in Fig S2B. The magnitude of ∆Cp indicates the degree of mAb-induced conformational changes on FL-rCSP. We have also removed some of the more esoteric thermodynamics/biophysics background from the Results section and modified the language to make it more intelligible to the typical biologist/immunologist.**

8. Fig S3 axis label typo ‘enthalpty’

- **We have corrected this.**

9. I don’t think that the data shown in Fig S4A supports the conclusion stated in the figure title. Instead it shows that L15 & mAb 15 do not potentiate spz neutralization by CIS43 or 311. The conclusion stated by the title cannot be drawn without ‘L15 only’ and ‘mAb 15 only’ conditions.

- **We agree that the title of Fig S4 erroneously suggests that L15 and mAb15 have some neutralization on their own, which was not our intent. We amended the figure title to state that “L15 and mAb15 do not potentiate the SPZ neutralization of CIS43 and 311,” as suggested by the reviewer.**

10. Lines 282-288 make sense as an investigation of one possible explanation for why a synergistic effect wasn’t seen, but don’t quite ‘fit’ at this point in the narrative - they do not support the concluding sentence of the paragraph / subsection. I think they either need to come after that sentence, or the concluding sentence needs to be modified to encompass the additional information from 282-288.

- **We agree with the reviewer and have moved the concluding sentence in question to the previous paragraph and created a new concluding sentence for the data described in lines 282-288.**

11. Fig S4B needs explanation in the legend of what the reader is meant to draw from it – it makes sense when referred to from the results section, but does not link to the title of Fig S4, or panel S4A.

- **We agree with the reviewer that the relevance of Fig S4B is not apparent in the figure title and have amended the title to “mAb15 and L15 neither potentiate the SPZ neutralization nor antagonize the SPZ binding of CIS43 and 311.”**

12. Line 457 – make clear that the Fc was human rather than mouse IgG1 (as stated in discussion). Some further explanation of the expected ability of this Fc to recruit Fc-dependent effector functions in the in vivo studies would be helpful.

- **We have added a sentence in the Methods stating that the mAbs were expressed in a human IgG_1_ vector. We have measured the binding of the human IgG_1_ vector used in this study to various mouse Fc receptors (FcR) *in vitro* using BLI, which is shown in the figure below. This data indicates that the human IgG_1_ vector used in our study has µM affinity to mouse FcγR *in vitro* by BLI, suggesting that the mAbs used in this study should efficiently activate mouse FcR. These data are being used in a separate manuscript and are beyond the scope of this report.**

13. The logical flow of the manuscript is slightly disrupted by jump from focus on C-CSP to the investigation of combinations of anti-repeat mAbs. The finding of lack of in vivo synergy between pairs of antibodies which are competitive antagonists of each others’ binding is not very surprising. I therefore think the readability of the manuscript might be improved if the final section of the results was de-emphasized, e.g. by mentioning it briefly in the manuscript and moving the data & description to supplementary information.

- **We believe there is a lot of interest in the malaria vaccine field as to how antibodies that bind different PfCSP epitopes may interact. Thus, we presented a comprehensive analysis of combining two PfCSP antibodies against different epitopes the repeat region and/or C-CSP (i.e., the two domains present in RTS,S) to address this question. This is important data for both vaccine design and use of monoclonal antibodies against PfCSP. Based on the new *in vivo* data we have added showing the improved protection of combining passive immunization of PfCSP repeat mAbs with active immunization of R21 in mice, we believe it is important to keep these data on combinations of monoclonals not being additive in the main part of the paper. Furthermore, combining potent repeat mAbs is of high interest in the malaria antibody field, especially as two of the potent mAbs we tested (CIS43 and L9) are in active clinical development for malaria prophylaxis. In presenting these data at meetings (both within the malaria field and in the wider infectious diseases community), there is always a question as to whether we have considered combining CIS43 with L9 or other potent repeat mAbs to provide improved protection against malaria. Our data clearly shows that combining what are currently the most potent human PfCSP repeat mAbs in the literature likely will not provide improved protection. Furthermore, the addition of the R21+mAb combination data (Fig 7 and S6 Fig) suggests that polyclonal antibody responses against the repeats and C-CSP interact cooperatively with potent repeat mAbs (but not C-CSP mAbs) to protect against SPZ challenge, which emphasizes the dual focus of this paper on combining repeat and C-CSP-specific antibodies.**
